# CKD Urine Metabolomics: Modern Concepts and Approaches

**DOI:** 10.3390/pathophysiology30040033

**Published:** 2023-09-29

**Authors:** Elena Y. Danilova, Anna O. Maslova, Andrey N. Stavrianidi, Alexander E. Nosyrev, Larisa D. Maltseva, Olga L. Morozova

**Affiliations:** 1 Molecular Theranostics Institute, Biomedical Science and Technology Park, I.M. Sechenov First Moscow State Medical University (Sechenov University), 8 Trubetskaya ul, 119991 Moscow, Russianosyrev_a_e@staff.sechenov.ru (A.E.N.); 2 Department of Chemistry, M.V. Lomonosov Moscow State University, 1 Leninskiye Gory Str., 119991 Moscow, Russia; 3 Department of Pathophysiology, Institute of Biodesign and Modeling of Complex System, I.M. Sechenov First Moscow State Medical University (Sechenov University), 13-1 Nikitsky Boulevard, 119019 Moscow, Russia; maltseva_l_d@staff.sechenov.ru (L.D.M.);

**Keywords:** chronic kidney disease, metabolomic, biomarkers, mass spectrometry, nuclear magnetic resonance

## Abstract

One of the primary challenges regarding chronic kidney disease (CKD) diagnosis is the absence of reliable methods to detect early-stage kidney damage. A metabolomic approach is expected to broaden the current diagnostic modalities by enabling timely detection and making the prognosis more accurate. Analysis performed on urine has several advantages, such as the ease of collection using noninvasive methods and its lower protein and lipid content compared with other bodily fluids. This review highlights current trends in applied analytical methods, major discoveries concerning pathways, and investigated populations in the context of urine metabolomic research for CKD over the past five years. Also, we are presenting approaches, instrument upgrades, and sample preparation modifications that have improved the analytical parameters of methods. The onset of CKD leads to alterations in metabolism that are apparent in the molecular composition of urine. Recent works highlight the prevalence of alterations in the metabolic pathways related to the tricarboxylic acid cycle and amino acids. Including diverse patient cohorts, using numerous analytical techniques with modifications and the appropriate annotation and explanation of the discovered biomarkers will help develop effective diagnostic models for different subtypes of renal injury with clinical applications.

## 1. Introduction

Chronic kidney disease (CKD) is a pathophysiological condition in which kidney function gradually declines. Ongoing damage to kidney cells impairs blood filtration, eventually leading to a chronic and intractable form of CKD. This condition has been identified as a leading cause of death worldwide [1], and regardless of the etiology, the decline in kidney function significantly affects quality of life and increases the risk of mortality [2]. The wide spread of CKD and its negative impact on a patient’s life quality makes CKD a socially significant pathology [3]. Systematic reviews and population-based studies indicate that CKD affects 1% of the pediatric population and has a global prevalence of 11–13%, which exceeds that of diabetes mellitus [4]. Chronic kidney disease (CKD) can lead to a variety of complications. These include cardiovascular disease, electrolyte and acid-base disturbances, anemia, mineral and bone disease, and volume excess [5,6]. The incidence of subsequent renal replacement therapy in pediatric CKD practice varies from 15–30 patients per million children in Eastern Europe to 100 per million children in Finland and the USA. Despite the prevalence of renal pathologies, the diagnosis of CKD is often complicated by the limitations of current approaches and the “blind spot” phenomenon. Currently, CKD can only be diagnosed in its advanced stages because there are no reliable methods for detecting the early stages or the “blind spot” of ongoing kidney damage. Late diagnosis increases the risk of complications and reduces the effectiveness of treatment [7].

Currently, the diagnosis of “chronic kidney disease”, according to Kidney Disease: Improving Global Outcomes (KDIGO) [8], is usually based on the glomerular filtration rate (GFR), blood urea levels, urinary albumin/creatinine ratio (ACR), and serum creatinine (sCr). A rapid decline in GFR is a significant risk factor for adverse clinical outcomes, as well as for the development and progression of CKD [9]. The GFR can be calculated using both endogenous (e.g., creatinine and cystatin C) and exogenous glomerular filtration markers (e.g., inulin and 99mTc-diethylenetriaminepentaacetic acid (99mTc-DTPA)). The most frequently used estimated GFR (eGFR) is based on the concentration of creatinine or cystatin C in blood plasma [10]. The Chronic Kidney Disease Epidemiology Collaboration (CKD-EPI) formula is recommended for calculating the GFR. It considers elementary demographic parameters (sex, age, race, height and serum creatinine). Calculation of the GFR using the the CKD-EPI formula provides more accurate results comparable to data obtained from 99mTc-DTPA clearance measurements. Another indicator of kidney damage is the sCr, but it is associated with several well-recognized limitations [11]. Creatinine changes may not accurately reflect changes in renal function and may be affected by variables such as race, gender, muscle mass, hydration, and medications [12]. Due to the compensatory adaptive capacity of the kidney and the indirect correlation between serum creatinine and the eGFR, serum creatinine levels typically rise only in the presence of 50–75% renal parenchymal injury [13,14,15]. Even with significant tubular injury, serum creatinine levels may remain unchanged, particularly in patients with good baseline renal function [14]. In addition, serum creatinine levels may rise due to hypovolemia, the intake of nonsteroidal anti-inflammatory drugs, or other factors that decrease renal perfusion without causing direct kidney damage [13].

The research on a unique CKD marker or a group of such markers is carried out to address all these limitations of existing diagnostics. The first study on protein markers, namely kidney injury molecule 1 (KIM-1), neutrophil gelatinase-associated lipocalin (NGAL), and N-acetyl-beta-d-glucosaminidase (NAG), showed encouraging results regarding their diagnostic potential [16]. However, the most recent large national prospective study of the Chronic Renal Insufficiency Cohort (CRIC) showed no improvement in risk prediction with additional KIM-1, NGAL, and NAG analysis compared with traditional diagnostic methods [17].

The polyetiological nature of CKD may limit the effectiveness of single markers in diagnosis. However, metabolomic analysis can address this feature of CKD due to the adaptability of multiparametric marker profiles. This characteristic should help solve the problem of early CKD diagnosis and improve and develop therapies. The concept of analyzing a wide range of markers for diagnostic purposes in nephrology has been introduced before. Nevertheless, with the development of high-throughput analytical and computational techniques, it has experienced a rebirth in the omics approach. Metabolomics, a part of this technology, involves the simultaneous analysis of low molecular weight metabolites. The first publications in nephrology-related metabolomics date back to 2009, focusing on renal cancer and nephrotoxicity [18]. This was followed in 2010 by the application of the metabolomic approach to CKD [19]

Developing and implementing novel analytical methods for biological biomarkers can provide insight into the distinct characteristics of CKD mechanisms across cohorts. Recent research has revealed that various factors may influence these mechanisms. Although type 2 diabetes often leads to CKD [9,10], many studies in recent years aimed to identify other causes of the disease and diseases that correlate with the detection of renal dependence in patients. For instance, the prevalence of CKD in a population can be influenced by factors such as individuals’ quality of life [2,7], the ecological condition of the environment [10], and diet [11]. Due to the possible influence of environmental factors with different statistical weights, some researchers use a disease nomenclature differentiated by country or region [12,13]. Also, renal complications such as AKI, which may be considered a risk factor for the development of CKD, are prevalent among patients following hospitalization for COVID-19 [20].

In summary, since metabolomic approaches represent a promising direction and developing field in CKD diagnostics, our review aims to summarize the results of urine metabolomics studies over the last five years in patients with CKD for both adults and children and to outline the main analytical methods used for urine analysis in patients with CKD.

## 2. Analytical Methods in CKD Metabolomics Studies

Modern analytical methods improve the search for CKD markers and expand the range of biomarkers detected in biological samples. “Omics” technologies are part of a high-throughput approach that has emerged in the last two decades. The types of detected molecules form the “four big omics” (i.e., proteomics, genomics, transcriptomics, and metabolomics) [21]. Metabolomic analysis is an extended analysis of low molecular weight (80–1200 Da) metabolite compounds in various biological samples. Using advanced statistical processing, the metabolite data are linked to biological systems’ structures, functions, and dynamics. The significant feature of obtained metabolomic profiles is their contiguity to the phenotype. A fundamental understanding of the pathophysiological process in relation to the phenotype should also be helpful in the development of therapeutic and prognostic methods.

In general, metabolomic studies can be performed using targeted or non-targeted approaches [22,23]. The former is focused on the search and analysis of a preselected set of compounds, while the latter includes a broader range of metabolites. Recent trends lean toward the use of non-targeted methods coupled with machine learning [24], and thus this classification is not present in all works. Both targeted and non-targeted approaches aim to identify discrimination features that can distinguish studied sample groups. The results can be used to develop new diagnostic or staging tools, as well as to explore new hypotheses about the etiopathogenesis of the disease [25].

The first publications on diagnosing CKD using a metabolomic approach have been published in the last decade [19,26]. Since then, the instrumentation and methods have significantly evolved. This section aims to describe the analytical methods used in the past five years of CKD urine metabolomics works.

Several analytical methods are employed in urine metabolomics. The main ones are tandem mass spectrometry, direct injection mass spectrometry (DI-MS), and nuclear magnetic resonance (NMR). Mass spectrometry (MS) delivers higher numbers of detected metabolites and greater sensitivity. Conversely, NMR spectroscopy has lower sensitivity but offers good compound identification with further structural information.

### 2.1. NMR Spectroscopy

NMR analysis could be performed on different nuclei. Biological samples are usually analyzed on the isotopes of the most abundant elements in biomolecules: ^1^H, ^13^C, ^14^N, and ^35^P. One of the first works that showed a connection between the essential marker trimethylamine N-oxide (TMAO) and CKD used a combination of ^1^H-, ^13^-C-, and ^14^N-NMR spectroscopy [27]. But today, only ^1^H-NMR spectroscopy has gained popularity in urine metabolomics due to having the highest abundance of ^1^H in all metabolites of all other nuclei and, consequently, the highest signal intensity.

The analysis of urine samples by ^1^H NMR raises the problem of solvent signal suppression due to the high water content in the probe. The dilution grade in urine is higher than in serum and other biofluids; thus, water signals interfere more with metabolite spectra. Therefore, the matrix effect is crucial to overcome in urine NMR spectroscopy analysis. The most common suppression method in urine CKD metabolomics is presaturation [3,28,29,30,31,32,33], which narrows signals and enhances the sensitivity and resolution of the spectrum. Presaturation could precede water signal elimination from the spectra [34] but also could be performed exclusively. For instance, in some works, presaturation was carried out using a ^1^H-nuclear Overhauser effect spectroscopy (NOESY) experiment [31,32]. Water signal removal improves the detection of low-concentration metabolites, and thus many works include this stage and often combine it with the Carr–Purcell–Meiboom–Gill (CPMG) spin-echo sequence [3,29]. In CKD, albuminuria is a common accompanying pathological condition. Thus, obstructing inhomogeneities caused by high protein content in a sample are also topical to overcome. CPMG minimizes the loss of phase coherence caused by inhomogeneities and removes the broad signals of the macromolecules, such as proteins and lipids.

Moreover, an additional increase in signal intensity can be achieved by the cryoprobe technique combined with transmit coil decoupling using inverse detection (TCI). The main advantage of cryoprobes is that they can significantly increase the sensitivity of NMR measurements by reducing thermal noise and improving the signal-to-noise ratio (SNR). Furthermore, TCI enhances the sensitivity and selectivity of the detection. A combination of a cryoprobe and TCI was applied in the Finnish Diabetic Nephropathy study [31] and an etiology-centered study of CKD [30].

Another feature of NMR studies is the dimension, or the number of independent variables measured during the experiment. Despite the multidimensional NMR resolving power, one-dimensional (1D) NMR with a NOESY-type pulse sequence is more prevalent as a faster and simpler experiment type [30,32,33,34]. Two-dimensional NMR (2D-NMR) was used in the CKD progression study [12], and the two-dimensional (2D) ^1^H J-resolved experiment was used for finding the biomarkers of both acute and chronic kidney disease [9].

Urine NMR analysis is a promising way to identify and analyze new marker molecules. The additional possibility to combine the identification of compounds of different classes creates great advantages for application in metabolomics research. The method’s modifications increase the detection’s resolution, sensitivity, and selectivity. However, there are disadvantages to the essence of the NMR method. For example, the high water content in urine samples requires specific steps for solvent suppression. It is also crucial to overcome matrix effects from peptides and other macromolecules.

### 2.2. Mass Spectrometry-Based Methods

Mass spectrometry-based methods comprise another common group of methods in metabolomic studies. The types of coupled MS could be divided into groups according to compound separation techniques. The most prevalent ones when considering types of metabolomics analysis are capillary electrophoresis (CE), gas chromatography (GC), and liquid chromatography (LC).

For analysis by gas chromatography-mass spectrometry (GC-MS), a sample must be prepared by being transferred to the gas phase through physical (micro solid-phase extraction from the vapor phase, evaporation, and steam extraction from the sample without additional concentration) or chemical (derivatization of compounds) methods. The chosen liquid-liquid extraction method without derivatization results in a limited number of volatile compounds, usually thermostable ones [35]. Clinical laboratories are much more likely to have access to tandem GC-MS. Therefore, the resulting panels of biomarkers may be easier to implement as an addition to existing diagnostic methods. The most robust approach in sample preparation could be performed if the headspace method was chosen. Headspace GC analyzes only the vapor phase above the sample; thus, the additional derivatization stage is unnecessary. Metabolite concentrations in the vapor phase are low, and thus high sensitivity and mass resolution time-of-flight mass spectrometry (TOF-MS) is chosen [36,37]. The derivatization step can also help overcome low sensitivity and enhance signals. The popular type of derivatization in urine metabolomics studies is trimethylsilyl derivatives [22,38]. They help improve the separation of metabolites, making detecting and quantifying them easier. Also, urease addition in sample pretreatment could be used [38].

In CKD metabolomic studies, the separation in the liquid phase could be performed by reversed-phase (RP) liquid chromatography-mass spectrometry (LC-MS), hydrophilic interaction liquid chromatography-mass spectrometry (HILIC-LC-MS), and capillary electrophoresis-mass spectrometry (CE-MS) methods. The two last approaches conform to the polar metabolites’ separation, while RP LC-MS is designed for nonpolar metabolite separation. There was no prevalence in any chromatography method in the considered metabolomic studies, as CKD’s markers belong to many different compound classes. Some studies combined RP-LC- and CE-MS [39] or RP- and HILIC-LC-MS [23] to increase metabolome coverage.

As in NMR metabolomic studies, a significant problem in method development is a high dilution of urine samples and, consequently, a low concentration of metabolites. To overcome this in tandem MS methods, the samples could be dried and reconstituted in a smaller amount of solvent [39,40], or solid-phase microextraction could be performed [41]. Furthermore, resolution and sensitivity enhancement could be achieved by different MS instrumentation. The prevalent amount of CKD metabolomic works were made using time-of-flight mass spectrometry (TOF-MS) [23,36,39,42] and quadrupole time-of-flight mass spectrometry (Q-TOF-MS) [43,44,45,46], which is characterized by high sensitivity, fast scanning speeds, and accurate mass measurements. However, in one of the works, both Q-TOF-MS and reconstitution techniques were performed [40]. While less popular, there are still other mass spectrometer types: the triple quadrupole mass spectrometer (TQ-MS) [23], Orbitrap MS [34], and Q-Exactive Focus mass spectrometer [47,48].

Also, the problem of a high protein presence in the urine of CKD patients was already mentioned. To overcome this, in tandem, MS or flow-injection MS methodic protein is precipitated by acetonitrile, methanol, or their water solutions being added in the sample, followed by centrifugation. To make the sample extra purified, the ultrafiltration stage is included in the sample preparation [42,47]. Some experiments consolidate the centrifugation and filtration stages using microspin filters [49].

Metabolomic studies, especially in large cohorts, require a robust and simple sample preparation method. The answer to this question was proposed in a “dilute-and-shoot” approach. The “dilute-and-shoot” approach in metabolomics has been used for many years and can be described as a specific type of sample preparation method in mass spectrometry-based metabolomics, where the sample is diluted and directly injected into the mass spectrometer without further cleanup or extraction steps. Kwan et al. used a method close to this approach in the CRIC study, which included 1001 participants altogether [44].

After the sample preparation and chromatographic separation steps, the MS detection stage occurs in tandem MS methods. In MS, molecules are identified and quantified by measuring the signal level of the mass-to-charge ratio of the ions (*m*/*z*) derived from those molecules. There are many techniques for producing ions in MS. Usually, in urine metabolomics, electrospray ionization (ESI) [42,43,47], chemical ionization (CI), and electron ionization (EI) [38] are present. One of the latest works in our scope used heated electrospray ionization (HESI) [48].

Compound identification complemented by databases (e.g., NIST) is the most typical approach for GC-MS experiments. In liquid chromatography, the identification of compounds is a more complex issue, and universal bases do not exist, as for GC. However, in some works, databases developed within the laboratory used research [43].

Another essential question is the verification of the obtained panel of markers. To solve this problem, a combination of different analytical approaches could be applied. For instance, the panel of markers combinates from both methods’ data, and then markers giving the statistically significant difference between groups are verified and chosen [3]. Another approach is applying a method combination relying mostly on their main advantages. Direct injection quadrupole time-of-flight mass spectrometry is a perfect method for untargeted marker searching. It could be combined with a more suitable method for target analysis CE-MS to approve the marker panel [50].

Mass spectrometry-based methods win over NMR in terms of sensitivity and are the perfect tools for metabolomic studies. GC-MS techniques may be easier to implement in clinical laboratories, but analyzing non-volatile metabolites could be associated with a time-consuming derivatization process. However, compound identification in MS analysis is not as straightforward as in NMR, and the high dilution factor also plays a role.

## 3. Biomarkers and Pathways

### 3.1. Pathogenesis of CKD

**Figure 1:**
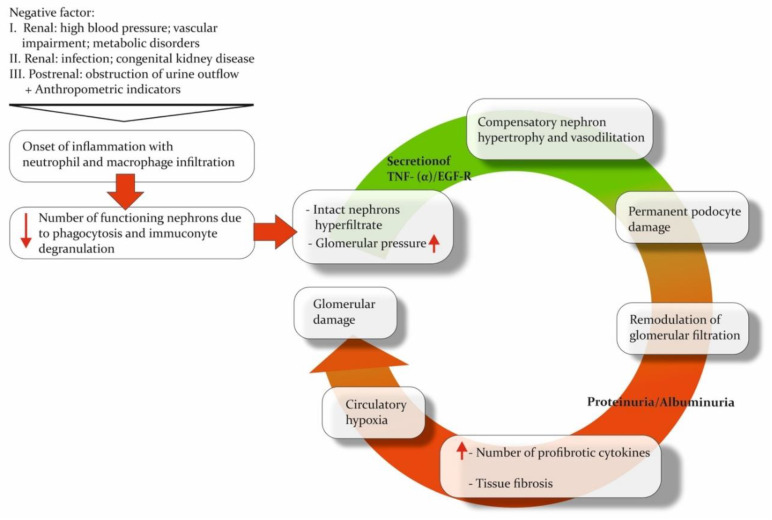
The “vicious circle” of CKD. Pathogenetic mechanisms underlying chronic kidney disease inevitably form a circle, leading to major organ damage. The direction of the red arrows shows the up- or down-regulation of processes or the increase/decrease in the number of cells or other functional units.

Regardless of the etiology of the damaging factor, CKD is based on similar pathogenetic mechanisms: loss of functional nephrons, damage to the convoluted tubules, inflammation, and fibrosis. An inflammatory reaction develops in the kidney parenchyma after exposure to any negative factor. This reaction leads to neutrophilic and macrophage infiltration and the proliferation of mesangial cells [51]. At the same time, the decrease in the number of functioning nephrons leads to the development of a hyperfiltration process in the intact nephrons. These changes compensate for the reduction in the number of active nephrons while at the same time significantly increasing the workload of the functioning nephrons [52]. The resulting raised glomerular pressure and hyperfiltration trigger the secretion of tumor necrosis factor-alpha/epithelial growth factor receptor (TNF-a/EGF-R), the activation of which induces nephron hypertrophy [52,53]. Nephron hypertrophy allows glomerular pressure to decrease by increasing the filtering area [18]. However, this could also result in damage to podocytes and remodeling of the filtration barrier, which raises its permeability and causes clinical manifestations of albuminuria or proteinuria [54]. Albumin and its complement pass through the filtration barrier, along with immune cells infiltrating damaged tissue. These processes stimulate the release of profibrotic cytokines by glomerular cells, leading to the maintenance of a pro-inflammatory microenvironment and the initiation of fibrosis [55,56,57,58]. The extensive growth of connective tissue leads to tissue and circulatory hypoxia, which increases damage to the glomerular and tubular apparatus of the kidney, ultimately completing the “vicious circle” of CKD pathogenesis (Figure 1) [59,60]. As a result, the CKD-induced processes in the renal parenchyma lead to its remodeling, namely metabolic changes, alterations in the quantitative and qualitative composition of proteins, and tissue structure modifications.

Changes in the composition of biological fluids and tissues provide an opportunity to use certain protein and non-protein molecules associated with specific pathways in the pathogenesis of CKD as biomarkers. In biopsy material [61,62,63] and, more commonly, in biological fluids, the concentration of biomarkers can be measured directly. Biofluids can reflect both the pathophysiological processes occurring directly in the kidneys and systemic changes [64].

Blood and urine can be collected in a sufficient volume for multiple tests simultaneously through minimally invasive techniques, offering a notable benefit to patients by alleviating discomfort associated with repeated collection of biomaterials and to researchers through the decreased cost of sampling procedures. Numerous studies have shown an association between the progression of CKD and the concentration of marker molecules in the blood or urine. These include structural protein (such as various types of collagens) biomarkers for tubular damage, inflammation, fibrosis, and hypoxia [65,66,67]. The studies related to molecular pathways allow us to categorize the features of the onset and stage of transformation of inflammation to CKD.

### 3.2. Markers of CKD

As previously mentioned, chronic kidney disease (CKD) is a polyetiological disease. The primary issue with CKD is that the inflammatory response and impaired renal function are accompanied by processes that partially sustain them. CKD is linked to various osmolytes and uremic solutes, which are associated with biochemical pathways in the body (Table 1). Uremic solutes were identified as potential markers in one of the earliest studies on CKD metabolomics.

Uremic solutes are toxic compounds. They are associated with uremia and subsequent changes in organ function. Robert D. Mair and colleagues performed urine metabolomic analysis by using LC-MS to study the clearance of uremic solutes [68]. However, this approach required urine and plasma samples to calculate the fractional clearance formula. Specifically, in patients with advanced disease, the most significant changes in clearance compared with control samples were observed for p-cresol sulfate, indoxyl sulfate, hippurate, and phenylacetylglutamine (Table 1). Alterations in the concentrations of these endogenous uremic solutes were the most significant findings. These changes are attributed to impaired protein binding of the solutes. Yet, the deterioration in uremic solute binding indicates impaired tubular secretion. The traditional GFR was affected less than the secretory clearance of solutes. Dried urine spot samples were also evaluated to detect changes in solute production, but no significant changes were observed [68].

This is consistent with the results of another study in which elevated indoxyl sulfate levels in urine were also correlated with elevated serotonin sulfate levels (Table 1). The authors suggested that both markers could be considered significant signals for the early diagnosis of CKD [69]. However, it is uncertain whether a single molecule can definitively resolve the issue of delayed CKD diagnosis. Targeting analysis of compounds related to indoxyl sulfate metabolism would be valuable to confirm these findings. Additionally, since this compound is classified as a toxin, it is unclear how early detection can be provided through this marker. We can hypothesize that the association between indoxyl sulfate and the microbiome in CKD may combine with TMAO to form markers of a common underlying cause. The association of metabolites with the microbiome is potentially problematic for early-stage CKD diagnosis [45]. However, such an assertion should be supported by evidence.

Osmolytes are the compounds involved in cellular osmotic regulation, which alters CKD. Ryan B. Gil and colleagues confirmed the altered concentration of uremic toxins (e.g., hippuric acid, indoxyl sulfate, and p-cresol sulfate) and included osmolytes in the developed diagnostic panel (Table 1) [28]. Two of the most important protective compounds from the osmotic stress effect are betaine and myo-inositol. These molecules showed the best prognostic value, also agreeing with eGFR. The confirming transcriptomic analysis accompanied these results on a murine CKD model. This is a great advantage of this work as it allows us to correlate the processes in the tissue with the obtained metabolomic models. Damage in the kidney tissue (e.g., microvasculature damage) relates to the impairment of tubular transport and renal medullary response. The main finding is that these abnormalities are the key processes reflecting osmolyte imbalance and thus CKD progression. Other metabolic pathways related to the eGFR slope are the tricarboxylic acid (TCA) cycle, lipid metabolism, and amino acid metabolism [28].

In addition to osmotic stress, kidney tissue is also affected by oxidative stress during the development of CKD. Markers of this process were observed in the Balkan Endemic Nephropathy (BEN) study. Additionally, the uremic toxin p-cresol was detected in the urine of a group of patients (Table 1). It was identified as the marker yielding the most statistically significant results for separating groups of patients and healthy individuals [35]. However, the preliminary nature of the findings represents a limitation of this work. We also recommend correlating these findings with results obtained from other analytical platforms to validate them.

Amino acids (AAs) undergo filtration and reabsorption in the kidney. Altered reabsorption of AAs in the proximal tubule may explain the increased urinary levels and decreased serum levels of AAs in patients with chronic kidney disease (CKD). With progressive renal dysfunction, the urinary levels of AAs increase, indicating impairment of both glomerular and tubular structures, even in the early stages of CKD. In the study by Galavan et al., phenylalanine was also included in the metabolomic profile of CKD [69]. This amino acid has been observed by researchers investigating different subtypes of CKD, which we will discuss below [29]. This can be explained by the data published this year on phenylalanine hydroxylase (PAH) activity. PAH is commonly found in the kidney, liver, and pancreas. According to Seojeong Kim, it has been shown that PAH may alter the immune system of the kidney tissue [70], which links these findings to the processes in the development of CKD. Nonetheless, urinary phenylalanine may function as an early indicator of CKD, as its concentration might be altered before the decrease in renal function.

**Table 1:** Markers of CKD. ꜛꜜ Increase and decrease in metabolite concentration, respectively.

References	Type of Marker	Pathway	Main Markers	Main Findings	Method	Population
[57]	Secretory clearance of waste solutes relative to the GFR	Not annotated to the pathway in the original work	Phenylacetylglutamine, p-cresol sulfate, indoxyl sulfate, hippurate	The traditional GFR values were altered less dramatically than secretory clearances for solutes in advanced CKD stages.	LC-MS	Patients with advanced CKD (*n* = 16, eGFR,12 mL/min per 1.73 m^2^) and control participants (*n* = 16)
[28]	Markers related to eGFR	Not annotated to the pathway in the original work	Uracil ꜜ, formic acid ꜜ, glycolic acid ꜜ, hippuric acid ꜜ	Elevated concentrations of myo-inositol and betaine are believed to be associated with the impairment of protein binding for solutes. Despite the reason, the decline in uremic solutes binding indicates impairment of tubular secretion.	^1^H-NMR	CKD patients (*n* = 227), a nested retrospective subgroup (*n* = 57)
		TCA cycle	Citric acid ꜜ			
		Amino acid metabolism	L-threonine ꜜ			
		Lipid metabolism	Ethanolamine ꜜ			
		The gut microbiome-derived uremic toxins	Indoxyl sulphate ꜜ, p-cresol sulphate ꜜ			
		Osmolyte transport	Myo-inositol ꜛ, betaine ꜛ			
	CKD prognostic markers	TCA cycle	Citric acid ꜜ			
		Lipid metabolism	Ethanolamine ꜜ			
		Not annotated to the pathway in the original work	Glycolic acid ꜜ, dimethylamine ꜜ, creatinine ꜜ, trimethylamine N-oxide ꜛ			
		Osmolyte transport	Myo-inositol ꜛ, betaine ꜛ			
[45]	Markers of CKD progression	TCA cycle	Glucose ꜛ	Since choline is often metabolized into other compounds, its urinary excretion is minimal, making its presence in a sample a precise indicator of kidney dysfunction and poor prognosis.	NMR, LC-MS for validation	789 patients with CKD (stage 1 = 340, stage 2 = 230, stage 3 = 219), 147 healthy control subjects
		Carbohydrate metabolism	Fumarate ꜛ, citrate ꜜ			
		Choline metabolism	Betaine ꜛ, choline ꜛ			
[69]	Markers related to eGFR	Not annotated to the pathway in the original work	Serotonin sulfate ꜛ, glycylprolylarginine, all-trans retinoic acid, methylarachidic acid	Both urinary and serum metabolomic profiles should be analyzed as dual variations in the metabolomic profile indicate both glomerular and tubular injury.	HPLC-QTOF- −MS (ESI+)	88 patients with CKD, staged by eGFR in 6 subgroups and 20 healthy control subjects
		Amino acid metabolism	Cysteine ꜛ, 5-methoxytryptohan ꜛ, phenylalanine ꜜ			
		Alcylcarnitines	Propenoylcarnitine, butenoylcarnitine ꜛ			
		Uremic toxins	Hippuric acid ꜛ, indoxyl sulfate ꜛ			
[71]	Markers of CKD during obesity	Uremic toxins	Hippuric acid ꜛ	High levels of hippuric acid found in the urine suggest an improved removal of uremic toxins after bariatric surgery.	GC-HRAM -MS	11 obese patients with CKD, 14 obese patients without CKD
		Amino acid metabolism	Valine ꜜ			
[35]	Markers of CKD and BEN	Uremic toxins	Phenolic compounds (including p-cresol ꜛ)	The presence of elevated p-cresol signals filtration injury, leading to p-cresol accumulation in the kidneys and, eventually, tissue inflammation.	GC-MS	35 healthy volunteers (53.9%), 25 BEN patients (38.5%), 5 CKD patients (7.7%)

### 3.3. Markers for CKD Subtypes

Diabetic kidney disease (DKD) is a highly prevalent form of CKD and is most commonly studied in the context of urinary metabolomics. However, several challenges are involved in formulating the format of a DKD study. Diabetes mellitus is a lifelong endocrine disease, and the manifestation of CKD can be detected at any time and at different rates. As a result, highly sensitive progression markers are essential.

Progression markers of DKD were examined in various experimental models. One approach involved annually evaluating the slope of a clinical parameter such as eGFR [22,39,44,50], time until the end stage [50], and time until incident kidney failure with replacement therapy [44]. An alternative method involved dividing the study cohort into groups based on the stage of DKD before comparing the samples [32]. Jing Zhang et al. studied the CRIC to detect and evaluate urinary markers of progression [50]. Every potential marker metabolite was analyzed with clinical factors in the discovery phase of the study. The results demonstrated that this approach is particularly important for evaluating the proximal outcome linked to the eGFR slope. The majority of pathways in the prognostic panel for end-stage kidney disease (ESKD) are related to amino acid metabolism. Tryptophan and branched-chain amino acid (BCAA) pathways are frequently included in final diagnostic panels for chronic kidney disease (CKD), and Jing Zhang et al. also endorsed them as ESKD risk markers for diabetic patients [50].

These results are consistent with the discovery of 3-hydroxyisobutyrate (3-HIBA) metabolite, which was identified as a catabolic intermediate of BCAA in a study of CRIC participants with diabetes [44]. Impairment in BCAA catabolism plays a significant role in the pathogenesis of type 2 diabetes and obesity [72]. In the work by Chasapi et al., 3-methyl-2-oxovalerate, a compound involved in BCAA catabolism, served as a diagnostic element to differentiate hypertensive nephrosclerosis from diabetic nephropathy [30]. The changes in the TCA cycle and BCAA catabolism could be associated with the decline of mitochondrial functions, angiogenesis, or the development of insulin resistance and ketoacidosis in diabetic patients [44]. However, in the non-diabetic cohort, both the individual and total levels of BCAAs showed a moderate probability of early-stage cardiovascular, metabolic, and renal disease, with the strongest likelihood being for the latter [49]. These data support the association of BCAA metabolism and catabolism with renal damage while highlighting its general basis.

But not only BCAA amino metabolism has been mentioned in recent works. Other amino acid pathways have been included in metabolomic diagnostic panels. Taherkhani et al. underlined the importance of aromatic amino acid metabolism in the early stage of contrast-induced nephropathy [38,73,74]. However, in the work of J.R. Lucio-Gutiérrez, et al., compounds related to amino acid metabolism and biosynthesis were associated with moderate and severe DKD [32].

The TCA cycle in mitochondria has adenosine triphosphate (ATP) production function and regulates glucose, fatty acid, and amino acid metabolism [74,75]. It was shown that deterioration in mitochondrial processes accompanies non-diabetic CKD [28] and DKD [22,32,44]. TCA cycle impairment could also be related to regulating inflammation and oxidative stress [73,74]. Nevertheless, Feng et al. showed that the simple diabetes mellitus group (SDM) exhibited a comparatively lower reduction in the concentration of compounds (participants of the TCA cycle) including citric acid, cis-aconitic acid, glycolic acid, and aconitic acid (produced) [47]. Albuminuria accompanies DKD in more than half of all cases. Albuminuria DKD (ADKD) cases are easier to diagnose. Although normoalbuminuric DKD (NADKD) is less common, it still leads to the same loss of kidney function. The increase in L-malic acid concentration, also involved in the TCA cycle, was found to be only in the NADKD group and not in ADKD [47]. Most researchers consider TCA metabolites to be significant potential indicators of kidney impairment [29,76]. We can hypothesize that differences in TCA cycle metabolites could be related to the variously modified mitochondrial function in CKD and its subtypes. Nevertheless, kidney disease’s metabolic profiles also reflect TCA’s association with fatty acid metabolism.

Compounds related to fatty acid metabolism are also common in DKD diagnostic panels (Table 2). Elevated albumin excretion and fatty acid loads filtrated by the glomerulus lead to the reabsorption of a bounded free acid surplus with albumin in the renal tubules [77]. Other processes connected with fatty acids’ metabolism and mentioned in the literature are kidney function maintenance [78], podocyte apoptosis by reactive oxygen species (ROS) formation [79], and keto acid metabolism [78]. Linoleic acid and γ-linolenic acid uremic concentrations have a dramatic difference in the ADKD group in comparison with the simple DM and NADKD groups [47]. Moreover, compounds related to fatty acid metabolism were included in the diagnostic panel, discriminating healthy people from the group with glomerulopathy. Ligor et al. underlined that detected ketones were the product of fatty acid oxidation and elevated levels of oxidative stress (Table 2). Furthermore, the authors proved that in addition to well-known biomarkers of kidney damage, such as cystatin C, NGAL, KIM-1, and interleukin 18, urine volatile organic compounds (VOCs) can be used in the diagnosis of glomerulopathies [32].

Another significant feature, as mentioned previously, is the rate at which chronic kidney disease (CKD) develops. Hirakawa et al. designed a marker panel for the rapid decline in DKD, using a combination of non-targeted metabolomics and advanced machine learning analysis [39]. These methods improved the data scope that could be extracted from patient samples. The urinary threonic acid was found to be a better marker of rapid decline in DKD. The role of threonic acid in pathogenesis is unclear due to the limited metabolomic panels reporting on it [39]. The study’s main strength is the high number of recruited patients, making the results especially significant. In another smaller cohort, some patients exhibited rapid progression of kidney dysfunction and changes in phospholipid metabolism. This could be due to the involvement of LPC (16:0 and 18:0) in the metabolic shift during DKD progression. The authors strengthened their results by conducting parallel in vitro studies on cultured proximal tubular cells. It demonstrated the cause of phospholipid markers in patients with a rapid decline in kidney function. The stress-induced, LPC-mediated lipotoxicity triggers organelle impairment and subsequent apoptosis. This phenomenon can serve as a potential target for future therapeutic interventions [46]. Thus, metabolomic analysis already appears to be able to discriminate patients predicted to progress to severe renal failure. We expect that further validation of prognostic markers in different cohorts will be required for full implementation.

Another important feature, as mentioned before, is the rate of CKD development. In the work of Hirakawa et al., a marker panel for the rapid decline in DKD was made by combining non-targeted metabolomics and comprehensive machine learning analysis [39]. Machine learning methods allowed improving the data scope that could be extracted from patients’ samples. Urinary threonic acid was a better marker of rapid decline in DKD. Threonic acid in pathogenesis is not clear because of limited metabolomic panels reported about it [39].

### 3.4. Markers of Acute Kidney Injury

Acute kidney injury (AKI) is defined as a rapid decline in kidney function. AKI can be viewed as a potential risk factor for the development of CKD and a consequence of CKD. However, AKI and CKD may coexist and interact in a bidirectional fashion. AKI can accelerate the progression of CKD, while CKD can increase one’s predisposition to AKI. This interaction can lead to a cycle in which AKI worsens CKD, increasing the risk of future AKI episodes. Both conditions may have similar symptoms, and diagnosing AKI can be as complex as diagnosing CKD.

Chen Chaoyi et al. found that a diagnostic panel for AKI that included xenobiotic metabolism played a critical role in disease manifestation (Table 3) [40]. Samples from healthy patients could be differentiated from those with AKI by identifying compounds related to xenobiotic metabolism, such as cytochrome P450 and arginine and proline metabolism [40].

Another method for studying the metabolomics of AKI was presented by Saito et al. The researchers analyzed samples from patients in an intensive care unit (ICU) 6, 12, 24, and 48 h after admission [42]. In the first model, it is assumed that the levels of metabolites are only influenced by the disease states: non-AKI, mild AKI, and AKI (as shown in Table 3). The second model assumes that disease states, time points, and subjects influence the metabolite levels. In the third model, changes in metabolite levels over time are assumed to be influenced by the disease state, in addition to the assumptions made in the second model. Unidentified metabolites were not included in this review. However, the authors suggested that they may have potential. One interesting finding was the downregulation of ethanolamine, which correlated with the 24 h time points for AKI patients. The researchers assumed this effect must be related to ROS downregulation [42]. 

### 3.5. Markers of Renal Impairment in Children

Chronic kidney disease (CKD) in children shares the same diagnostic problems but has additional limitations in treatment and transplantation. However, research on pediatric cohorts is limited. In papers describing metabolomic profiles in children, the number of samples is exceptionally small, and population studies are lacking. We believe this allows us to characterize the results presented below as preliminary.

Diagnostic metabolomic panels typically include markers associated with the TCA cycle and amino acid and fatty acid metabolism, as noted above. This is also true for studies based on the results of pediatric cohort analyses (Table 4) [23,33,43,84,85].

Maciozsek et al. performed a metabolomic analysis to investigate significant variations in biochemical pathways in children who had been diagnosed with renal dysplasia [23]. The results showed a decrease in the levels of acylcarnitines, indoxyl sulfate, xanthine, glutamine, and aconitate, accompanied by increased levels of lactate, dimethylguanosine, and guanidinosuccinate in the patients’ urine. Based on the collected data, the authors concluded that the regulatory processes of glycolysis (the Warburg effect), the ornithine cycle, the tricarboxylic acid cycle, purine metabolism, and fatty acid biosynthesis are disrupted in children with renal dysplasia [23]. The research conducted by Franiek et al. analyzed non-volatile compounds and revealed a metabolomic classifier involving homovanillic acid, taurine, glutamine, methionine, aspartic acid, histidine, and kynurenine, which are associated with amino acid metabolism (Table 4) [84]. The classifier’s nine metabolites have been previously studied. The changes in their concentrations in this study’s samples can be attributed to glomerular inflammation, osmotic imbalance in the tissues, and oxidative stress [84].

The results of studies on pediatric samples are not yet sufficient to show clear differences in metabolomic panels for different age groups. However, in addition to the obvious physiological differences, it is important to note that the pathologies leading to CKD are also different. For instance, standing alone are studies focusing on the metabolome of neonatal patients. Technical abbreviations will be explained upon first usage. Scalabre et al. evaluated the diagnostic potential of metabolomics in identifying unilateral renal pelvis dilatation (RPD) and ureteropelvic junction obstruction (UPJO) in newborns [33]. The group of samples from newborns with RPD related to UPJO can be distinguished from the control group based on a reduction in the concentrations of betaine, creatine, threitol, glucuronate, alanine, arginine, lysine, threonine, N, N-dimethylaniline, ornithine, taurine, and TMAO [33]. Studies combining a metabolomic approach and cohorts of patients younger than one year are extremely rare. Therefore, it is difficult to correlate these results and to speak on general trends. In our opinion, it would be promising to repeat the experiment with a more sensitive MS method in order to confirm the panel of markers obtained.

## 4. Conclusions

CKD is a challenging disease. Because of its specific pathological course, inflammation that starts in one part of the organ gradually spreads to functional kidney tissues. The existing diagnostic methods are insufficient, resulting in a delay in detecting this process. However, there is a promising opportunity for diagnostic method development based on a metabolomic approach. Typically, urinary metabolic studies in CKD patients utilize NMR and tandem MS techniques. However, not every medical center is equipped to perform analysis using expensive MS and NMR analyzers. These instruments’ limited availability and high cost hamper the widespread use of metabolomic screening methods. This poses a challenge in developing and optimizing methods that align with the specific characteristics of the clinical laboratory. In addition, matrix effects and high initial sample dilution must be taken into account when developing urinalysis methods for metabolomics.

In recent years, several studies have illustrated the potential for creating diagnostic profiles for various stages of CKD, nephropathy types, and risk assessment. Furthermore, this study demonstrates the potential to create diagnostic profiles for the normoalbuminuric form of CKD and renal dysplasia independent of eGFR. The metabolomic approach is promising and can contribute to the development of personalized medicine. However, the reviewed studies have limitations in terms of the tentative discriminative power of metabolomic markers.

The compounds identified as markers span several classes, and their diversity is apparent for both their age groups and similar pathologies. Furthermore, different studies showed multidirectional variation in the concentration of the same compound for the groups of samples studied. Therefore, it is necessary to further validate potential metabolites for the development of comprehensive metabolomic panels. Conducting studies comparing urine analysis results from multiple cohorts using the same approaches is highly promising. While no fixed set of compounds is consistently found across studies, there are consistent findings related to changes in urinary composition caused by mitochondrial dysfunction, oxidative stress, membrane dysfunction, and metabolic abnormalities. TCA, amino acid, and fatty acid metabolism abnormalities have also been demonstrated. The connection between CKD and the microbiome has been noted as well. Rather than focusing on individual metabolic markers, analysis of groups of compounds involved in specific pathways and processes may provide a solution to the diagnostic challenge.

The metabolomic approach presents vast potential for advancing personalized medicine related to CKD. However, additional research is needed to address the challenges of cost, availability, validation, and standardization of urine metabolomic analysis to maximize the benefits of metabolomics in the diagnosis and prognosis of CKD.

## Figures and Tables

**Table 2:** Markers of DKD. ꜛꜜ Increase and decrease in metabolite concentration, respectively.

References	Type of Markers	Pathway	Main Markers	Main Findings	Method	Population
[34]	Markers of rapid decline in DKD	Not annotated to the pathway in the original work	1-methyl pyridine-1-ium (NMP), retinol-1, trigonelline, threonic acid, ethanolamine, choline, 4-(trimethylammonio) but-2-enoate (CE-C-0218)	Non-targeted metabolomics and comprehensive machine learning analysis allowed us to reveal important features in DKD progression prediction.	CE-TOF–MS, LC-TOF–MS	Group 1 (n = 46) eGFR change rate was above 0%; group 2 (n = 34) below 0% and above −3.3%; group 3 (n = 39) below −3.3% and above −10%; group 4 (n = 14) <10% (rapid decliner)
[39]	DKD progression prospectively for a median of 8 (range: 2–10) years	TCA	Citric acid, aconitic acid	Obtained an extended panel of progression markers correlated with previous studies. Metabolites of TCA and BCAA catabolism are linked to the deterioration of mitochondrial functions and angiogenesis or insulin resistance and ketoacidosis.	FI-MS (Q-TOF)	1001 Chronic Renal Insufficiency Cohort (CRIC) participants with diabetes
		Catabolic intermediate of branched-chain amino acid (BCAA)	3-hydroxyisobutyrate (3-HIBA), tiglylglycine			
		Not annotated to the pathway in the original work	Uracil, glycolic acid, 3-methyladipic acid, homovanillic acid, aconitic acid, 3-hydroxypropionate, 2-methylacetoacetate, 2-ethyl-3-hydroxypropionate			
[43]	Metabolomic profiles and markers of progression	Not annotated to the pathway in the original work	C10:3, acyl-carnitine	The study found that higher levels of tryptophan were associated with higher end-stage kidney disease (ESKD) risk in both untargeted and targeted analyses, and the tryptophan pathway was significantly enriched in a set of ESKD-related metabolites.	FI-Q-TOF-MS (untargeted), CE-MS (target)	995 randomly selected CRIC participants with diabetes across CKD
		Amino acid metabolism	Tryptophan, valine, asparaginyl-hydroxyproline, arginyl glutamine			
		Food or drug derivatives	3-(4-methyl-3-pentenyl) thiophene			
[40]	Albuminuria DKD (ADKD) difference from normoalbuminuric DKD (NADKD)	TCA cycle	L-malic acid ꜜ	Metabolites related to the linoleic acid metabolism, citrate cycle, and arginine and proline metabolism allowed differentiating the ADKD group from the SDM and NADKD groups but not between the SDM and NADKD groups.	UPLC–MS/MS	SDM group (UACR < 30 mg/g and eGFR ≥ 90 mL/min/1.73 m^2^, n = 30); ADKD (30 ≤ UACR < 300 mg/g and eGFR ≥ 45 mL/min/1.73 m^2^, n = 30); NADKD (DKD patients with UACR < 30 mg/g and 45 ≤ eGFR < 90 mL/min/1.73 m^2^, n = 35)
		Arginine and proline metabolism	L-proline ꜜ, L-erythro-4-hydroxyglutamate ꜜ, spermidine ꜜ			
		Linoleic acid metabolism	Linoleic acid ꜛ, γ-linolenic acid ꜛ			
	Albuminuria DKD (ADKD) difference from simple diabetes mellitus group (SDM)	TCA cycle	Succinic acid ꜜ, cis-aconitic acid ꜜ, citric acid ꜜ			
		Arginine and proline metabolism	L-proline ꜜ, L-erythro-4-hydroxyglutamate ꜜ, N-methylhydantoin ꜜ, N-carbamoyl putrescine ꜜ, spermidine ꜜ, 5-aminopentanoic acid ꜜ			
		Linoleic acid metabolism	γ-linolenic acid ꜛ			
[29]	DM2-CKD mild	Not annotated to the pathway in the original work	Alanine ꜛ, 2-hydroxybutyrate ꜛ	The groups of metabolites were significantly different in patients with mild, moderate, and severe CKD. An increase in trigonelline in DM2 patients due to creatinine depletion was found for the first time. 2-hydroxybutyrate urinary consent correlated with the body mass index.	1D NOESY ^1^H -NMR	Control (n = 17), DM2 (n = 6), DM2-CKD mild (n = 13), DM2-CKD moderate (n = 10), DM2-CKD severe (n = 14)
	DM2-CKD mild	Glyoxylate, dicarboxylate metabolism	Citrate ꜛ			
	DM2-CKD moderate	Amino acid metabolism	Hippurate ꜛ			
	DM2-CKD severe	Glycolysis and gluconeogenesis, pyruvate metabolism	Lactate ꜛ			
	DM2-CKD severe	Glyoxylate and dicarboxylate metabolism	Glycolate ꜜ			
	DM2-CKD moderate and severe	Amino acid metabolism and biosynthesis	Phenylalanine ꜛ			
	DM2-CKD moderate and severe	Not annotated to the pathway in the original work	Trigonelline ꜛ			
[76]	Markers of DKD	Monosaccharide and TCA cycle	Citrate, mannose ꜛ	Based on our multidisciplinary analysis, urinary myo-inositol concentration can increase predictive power when used in combination with serum creatinine and UPCR in ESRD progression.	Targeted NMR	Patients with DKD stages 1–5 (n = 208) and healthy controls (n = 26)
		Not annotated to the pathway in the original work	Myo-inositol ꜛ, choline			
[80]	Markers of DKD	Amino acid metabolism	Arginine ꜛ, citrulline ꜛ, ornithine ꜛ	Acylcarnitines are more sensitive markers of early diabetic kidney failure in type 2 diabetes in patients with normoalbuminuria and microalbuminuria than Hb1Ac. They interact with NF-Kβ, initiating inflammation and insulin resistance.	Targeted GC/MS	232 patients with type 2 diabetes mellitus and 150 healthy controls
		Alcylcarnitines	Dodecanoylcarnitines C12, triglylcarnitine C5:1, isovalerylcarnitine C5			
[81]	Markers related to Immunoglobulin A nephropathy progression	Aminoacyl-transfer RNA biosynthesis	Glutamine Valine Leucine Tyrosine	The prediction of IgAN progression improved significantly when proteinuria was combined with serum glycerol/threonine or urine leucine-valine.	NMR	Non-progressors, progressors, healthy control (n = 10 for each group)
		Valine, leucine, and isoleucine biosynthesis	Leucine, valine ꜛ			
		TCA cycle intermediates	D-glucose ꜛ, sucrose ꜛ, gluconic acid ꜛ, l-xylonate-2, oxalic acid			
[82]	Markers of early DKD stages	Not annotated to the pathway in the original work	o-phosphothreonine, aspartic acid, 5-hydroxy lysine, uric acid, methoxytryptophan	The discovery of these candidate biomarkers implies their contribution to early DKD and 2DM advancement. This is because, even in the early stages of DKD, it can indicate kidney damage at specific sites along the nephron.	UPLC-QTOF-ESI* MS	90 patients with type 2 DM, classified into three subgroups according to albuminuria stage from P1 to P3 (30 normo-, 30 micro-, and 30 macroalbuminuric) and 20 healthy controls
[83]	Markers related to early immunoglobulin A nephropathy	Amino acid metabolism	Glycine ꜛ	A high glycine concentration could potentially ameliorate the inflammatory damage induced by TNF-alpha. The activation of the tubules in IgAN due to glomerulotubular communication could be addressed by glycine. Physiological changes in renal tubular metabolism could increase glycine levels. IgAN patients have a significant reduction in protein H, which forms the glycine cleavage system, but without reduced eGFR.	NMR	Membranous nephropathy (MN) patients (n = 81), minimal change disease (MCD) (n = 49), lupus nephritis (LN) (n = 38) patients, and healthy controls (n = 146)
[46]	Markers of DKD’s fast decline	Phospholipid metabolism	Lysophosphatidylcholine ꜛ (16:0 and 18:0)	The accumulation of these compounds results from impaired lipid metabolism and leads to oxidative stress of organelles and apoptosis through the PPARd-PLIN2 pathway.	MS	150 patients with stage G3 DKD
[18]	Risk prediction of CKD progression in individuals with type 2 diabetes mellitus (T2DM)	TCA	Lactate ꜛ, malate ꜛ, fumarate ꜛ, citrate ꜜ	Oxidative stress in CKD progression is connected with fumarate production. Fumarate and malate could be predictors of CKD progression independent of traditional cardio-renal risk factors.	GC-MS (selected ion monitoring)	Discovery study: progressors (n = 116), non-progressors (n = 271); validation study: progressors (n = 96), non-progressors (n = 402)

**Table 3:** Markers of AKI. ꜛꜜ Increase and decrease in metabolite concentration, respectively.

References	Type of Markers	Pathway	Main Markers	Main Findings	Method	Population
[35]	AKI diagnostic markers	Metabolism of xenobiotics by cytochrome P450	2-S-glutathionyl acetate	Characteristic AKI metabolomic markers were mainly related to xenobiotic, taurine, and hypotaurine metabolism.	UPLC–MS (Q/TOF)	AKI (*n* = 30) and healthy controls (*n* = 20)
		Taurine and hypotaurine metabolism	5-l-glutamyl-taurine			
		Metabolism of xenobiotics by arginine and proline metabolism	l-phosphoarginine			
[37]	AKI after invasive surgery. Model 1	Not annotated to the pathway in the original work	Ethanolamine ꜜ, glutamine ꜜ, glycine ꜜ, 2-hydroxypentanoate, serine, succinate	The study identified AKI-specific metabolites and time points, which may lead to improved biomarker development.	CE-TOF-MS	Non-AKI: 23, mild AKI: 24, severe AKI: 14 were measured, followed by the measurement of urine samples from 60 additional patients (non-AKI: 40, mild AKI: 20)
	AKI after invasive surgery. Model 2		Glycine ꜜ, urea, urate, ethanolamine ꜜ, glutamine, N, N-dimethylglycine			
	AKI after invasive surgery. Model 3		Piperidine, taurine, methanesulfonate, 3-hydroxykynurenine			

**Table 4:** Markers of renal impairment in children. ꜛꜜ Increase and decrease in metabolite concentration, respectively.

References	Type of Markers	Pathway	Main Markers	Main Findings	Method	Population
[66]	Metabolites overlapped between the pre-AKI and AKI panels	Amino acid metabolism	Taurine, glutamine, methionine, aspartic acid, histidine, kynurenine *ꜛ*	Inflammation of renal cells leads to disruption of membrane integrity and tubule apoptosis in AKI, activation of compensatory functions, and loss of maintaining an osmotic balance. Renoprotective biomarkers are clusterin and cystatin C. Impaired renal function also affects kidney blood flow and vascular endothelial function.	GC-MS, direct flow injection MS (DI-MS)	Pre-AKI (*n* = 15), AKI (*n* = 22), and respective controls (*n* = 30)
		Amino acid metabolism, catecholamine metabolism	Homovanillic acid			
		Components of the lipid bilayer	Phosphatidylcholine (PC.aa.C34.1), sphingolipid (SM.C16.0)			
	Pre-AKI	Not annotated to the pathway in the original work	Acylcarnitines (C5.DC.C6.OH., C2,C7.DC,C9,C3.DC.C4.OH.), phosphatidylcholine (PC.aa.C36.1)			
		Not annotated to the pathway in the original work	Acetylornithine, serotonin, arginine, methylmalonic acid			
[38]	Acute kidney injury 24 h after the diagnosis of sepsis	Amino acid metabolism	Histidine	An effective diagnostic panel of markers for SA-AKI was demonstrated. Glycerophospholipid metabolism is related to the pathophysiology of septic AKI.	UPLC HILIC -QTOF/MS, Triple TOF	Septic children with AKI (*n* = 27) and septic children without AKI (*n* = 30)
		Tyrosine metabolism, ascorbate and aldarate metabolism	Gentisaldehyde, 3-ureidopropionate, N4-acetylcytidine, and 3-methoxy-4-hydroxyphenylglycol sulfate			
	Acute kidney injury 12 h ALL after the diagnosis of sepsis	N-galactose metabolism, fructose and mannose metabolism, glyoxylate and dicarboxylate metabolism, β -alanine metabolism, and glycerophospholipid metabolism	L-histidine, DL-indole-3-lactic ac id, trimethylamine N-oxide, and caprylic acid			
		TCA compensation	L-glutamine			
[67]	Biomarkers for ureteropelvic junction obstruction (UPJO)	Amino acid metabolism	Alanine *ꜜ*, arginine *ꜜ*, lysine *ꜜ*, threonine *ꜜ*, N,N-dimethylaniline *ꜜ*, taurine *ꜜ*, ornithine *ꜜ*	Found diagnostic biomarkers of UPJO and an early-stage transient dilatation demonstrated promising results and allowed differentiating all groups.	^1^H-NMR	Newborns with prenatally diagnosed RPD (*n* = 50), healthy newborn controls (*n* = 90)
		Betaine metabolism	Betaine *ꜜ*			
		Not annotated to the pathway in the original work	Creatine *ꜜ*, threitol *ꜜ*, glucoronate *ꜜ*			
[30]	Differentiating metabolites of established AKI patients, healthy and hospitalized patients without AKI	Amino acid metabolism	Leucine *ꜛ*, valine *ꜛ*	The decrease in the concentration of TCAs suggests that the observed effect originates from tubular cell membrane dysfunction connected with the transcellular transport of dicarbonic acids.	^1^H-NMR	65 neonatal and pediatric patients with established AKI of heterogeneous etiology; healthy children (*n* = 53); group of critically ill children without AKI (*n* = 31)
		TCA cycle	Citrate *ꜜ*			
		Not annotated to the pathway in the original work	Bile acid *ꜛ*			
[19]	Metabolic signature of renal dysplasia, unrelated to eGFR value	Amino acid metabolism	Indoxyl sulfate *ꜜ*, glutamine *ꜜ*, glyceric acid *ꜛ*	The authors suggested that decreased acylcarnitine concentrations could indirectly indicate impaired mitochondrial function. This may also cause abnormalities in oxidative phosphorylation and fatty acid oxidation. These findings are consistent with cellular processes characteristic of CKD, such as ATP depletion, apoptosis, cell dedifferentiation, and intracellular lipid deposition.	GC-MS (GC-QQQ/MS), LC-TOF-MS (RP; HILIC)	72 children: renal dysplasia (*n* = 39, mean age of 5.68 years (range: 0.08–17.40)) and healthy controls (*n* = 33, mean age of 7.28 years (range: 0.09–17.69))
		Purine metabolism	Xanthine *ꜜ*			
		Fatty acid metabolism and biosynthesis	Acylcarnitines, hexadecanoic acid *ꜛ*			
		TCA	Aconitate *ꜜ*			
		Carbohydrates metabolism and biosynthesis	Arabitol *ꜛ*, lactose *ꜛ*, lactic acid *ꜛ*			
		Microbial metabolism	Furoic acid *ꜛ*			
		Ascorbate and aldarate metabolism	Threonic acid *ꜛ*			
		tRNA degradation	Dimethylguanosine *ꜛ*			

## Data Availability

Not applicable.

## List of Abbreviations

3-HIBA	3-hydroxyisobutyrate
99mTc-DTPA	99mTc-diethylenetriaminepentaacetic acid
ACR	urinary albumin/creatinine ratio
ADKD	albuminuria DKD
AKI	acute kidney injury
ATP	adenosine triphosphate
BCAA	branched-chain amino acid
BEN	Balkan Endemic Nephropathy
CE	capillary electrophoresis
CE-MS	capillary electrophoresis mass spectrometry
CI	chemical ionization
CKD	chronic kidney disease
CKD-EPI	chronic kidney disease epidemiology collaboration
CRIC	chronic renal insufficiency cohort
CPMG	Carr–Purcell–Meiboom–Gill
DI-MS	direct injection mass spectrometry
DKD	diabetic kidney disease
EI	electron ionization
eGFR	estimated glomerular filtration rate
ESI	electrospray ionization
ESKD	end-stage kidney disease
GC	gas chromatography
GC-MS	gas chromatography-mass spectrometry
GFR	glomerular filtration rate
HESI	heated electrospray ionization
HILIC-LC-MS	hydrophilic interaction liquid chromatography-mass spectrometry
ICU	intensive care unit
IL-18	interleukin-18
KDIGO	Kidney Disease: Improving Global Outcomes
KIM-1	kidney injury molecule 1
LC	liquid chromatography
LC-MS	liquid chromatography-mass spectrometry
MS	mass spectrometry
NADKD	normoalbuminuric DKD
NAG	N-acetyl-beta-d-glucosaminidase
NGAL	neutrophil gelatinase-associated lipocalin
NIST	National Institute of Standards and Technology
NMR	nuclear magnetic resonance
NOESY	nuclear Overhauser effect spectroscopy
PAH	phenylalanine hydroxylase
Q-TOF-MS	quadrupole time-of-flight mass spectrometry
ROS	reactive oxygen species
RP	reverse phase
RP LC-MS	reverse-phase chromatography-mass spectrometry
RPD	renal pelvis dilatation
sCr	serum creatinine
SDM	simple diabetes mellitus group
SNR	signal-to-noise ratio
TCA cycle	tricarboxylic acid cycle
TCI	transmit coil decoupling using inverse detection
TMAO	trimethylamine N-oxide
TNF-a/EGF-R	tumor necrosis factor-alpha/epithelial growth factor receptor
TOF-MS	time-of-flight mass spectrometry
TQ-MS	triple quadrupole mass-spectrometer
UPJO	ureteropelvic junction obstruction
VOCs	volatile organic compounds
